# Non-Invasive Hemodynamic Monitoring in Critically Ill Patients: A Guide for Emergency Physicians

**DOI:** 10.3390/jcm14197002

**Published:** 2025-10-03

**Authors:** Michela Beltrame, Mattia Bellan, Filippo Patrucco, Francesco Gavelli

**Affiliations:** 1 Emergency Medicine Department, “Azienda Ospedaliero Universitaria Maggiore della Carità”, 28100 Novara, Italy; 2 Department of Translational Medicine, Università degli Studi del Piemonte Orientale, 28100 Novara, Italy; 3 Division of Respiratory Diseases, “Azienda Ospedaliero Universitaria Maggiore della Carità”, 28100 Novara, Italy

**Keywords:** hemodynamic monitoring, non-invasive cardiac output measurement, fluid responsiveness, emergency medicine, shock resuscitation

## Abstract

Hemodynamic monitoring is fundamental in the management of critically ill patients with acute circulatory failure. The invasiveness of conventional devices, however, often limits their applicability in the emergency department (ED). Recent advances have introduced non-invasive modalities (including echocardiography, bioreactance, and plethysmography) that extend the use of hemodynamic assessment beyond the intensive care unit. Among various available techniques, bedside ultrasound (Point-of-Care Ultrasound, POCUS) emerges as a particularly versatile tool for rapid and comprehensive assessment of cardiac function and volume status. When integrated with continuous technologies such as bioreactance or pulse contour analysis, it allows for the adoption of more dynamic and personalized fluid management strategies. Currently, a multimodal and patient-centered approach represents the most effective paradigm for non-invasive hemodynamic evaluation in the emergency setting. This strategy enhances diagnostic accuracy and enables timely interventions guided by pathophysiological principles. Despite the inherent limitations of each technique, their integration provides emergency physicians with real-time information, with potential benefits on clinical outcomes and resource utilization. This review aims to outline the pathophysiological rationale for adopting non-invasive monitoring in the ED and to critically evaluate the advantages and limitations of each technique, providing emergency physicians with a concise framework to guide clinical practice.

## 1. Introduction

In the context of acute circulatory failure—such as hypovolemic or septic shock—fluid administration constitutes a cornerstone of early hemodynamic management. The primary goal is to enhance tissue oxygenation and organ perfusion by increasing cardiac output (CO) [1]. This can be achieved by augmenting the mean systemic filling pressure, thereby improving venous return and subsequently increasing CO. However, the relationship between fluid administration and hemodynamic response is not linear. Although fluid therapy may improve microvascular perfusion and tissue oxygenation, these effects are neither consistent nor universally observed [2].

**Figure 1:**
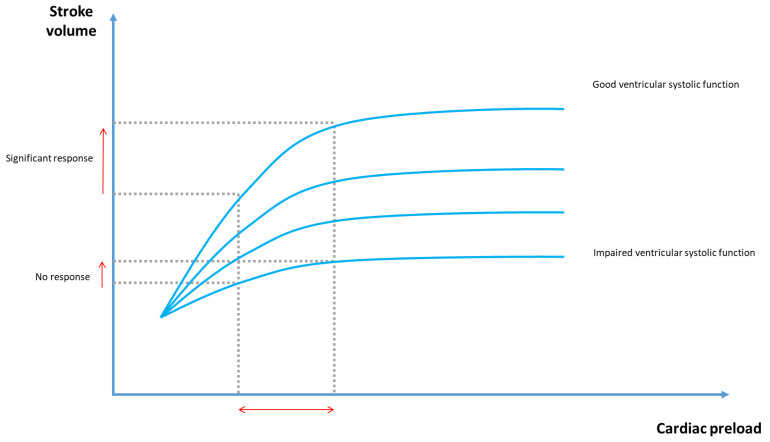
Relationship between Cardiac Preload and Stroke Volume According to the Frank–Starling Law.

When both ventricles operate on the steep portion of the Frank–Starling curve (Figure 1), an increase in preload leads to a rise in stroke volume (SV) and, consequently, in CO, potentially improving oxygen delivery [3]. According to this principle, myocardial contractility increases with greater initial fiber length (i.e., preload), up to a physiological limit. Beyond this threshold, additional intravascular volume expansion fails to further increase SV or CO and may instead become harmful by raising filling pressures and contributing to fluid overload [3]. In other words, when the heart operates on the flat, terminal portion of the Frank–Starling curve, further increases in preload no longer result in meaningful improvements in CO. This condition is known as preload-independence [4]. Under these circumstances, continuing fluid expansion may become harmful.

Fluid overload can lead to interstitial and pulmonary edema, impair gas exchange and cause hemodilution. Moreover, it may reduce pulmonary compliance, increase the work of breathing, and elevate intra-abdominal pressure. These effects can compromise hepatic and renal perfusion and negatively affect clinical outcomes [5].

Given these risks, clinical assessment alone is insufficient to guide fluid therapy. Instead, hemodynamic monitoring tools are essential for assessing preload responsiveness and tailoring fluid administration to the individual patient’s physiological status. As emphasized in recent literature, fluids should be regarded as pharmacological agents: carefully dosed, titrated, and continuously reassessed [6].

## 2. Physiological Concepts in Hemodynamic Monitoring

To understand the hemodynamic alterations and compensatory mechanisms of the macrocirculation, it is essential to introduce foundational concepts related to the cardiac cycle and key hemodynamic parameters.

The cardiac cycle consists of alternating phases of systole (ventricular contraction) and diastole (ventricular relaxation), which generate rhythmic pressure and volume changes within the heart chambers. Important volumetric parameters include:

- End-Diastolic Volume (EDV): the maximum ventricular volume at end of diastole, before ejection;
- End-Systolic Volume (ESV): the residual volume remaining after systole;
- Stroke Volume: the amount of blood ejected per heartbeat, calculated as SV = EDV − ESV [7].

Cardiac Output quantifies the total blood volume pumped per minute and is determined by the product of stroke volume and heart rate (HR):

CO=SV×HR

To account for differences in body size, CO can be normalized to body surface area and expressed as the Cardiac Index (CI), with a normal value of ~3.5 ± 0.7 L/min/m^2^ [7].

CO is dynamic and modulated by both heart rate and stroke volume. Heart rate is primarily governed by autonomic nervous system input, while stroke volume is influenced by three principal determinants: preload (EDV), myocardial contractility, and afterload [7]. According to the Frank–Starling mechanism, increasing preload stretches myocardial fibers, thereby enhancing contractile force, up to a physiological limit. Beyond this threshold, further increases in preload result in ventricular dilation without a corresponding increase in output. This relationship is further informed by Laplace’s law, which states that wall stress is inversely related to wall thickness and directly related to chamber radius. Thus, smaller or thicker ventricles generate less wall stress for a given pressure [7].

Mean Arterial Pressure (MAP) serves as a surrogate for tissue perfusion pressure and should ideally be maintained above 65 mmHg to prevent organ hypoperfusion [8,9]. In patients with chronic hypertension patients, however, higher MAP targets (80–85 mmHg) may be beneficial [10,11]. MAP is commonly estimated using the formula:

MAP=(SBP+2×DBP)/3

Pulse Pressure (PP), defined as PP = SBP − DBP, reflects SV and is inversely related to arterial compliance [12]. Variations in pulse pressure during mechanical ventilation—termed Pulse Pressure Variation (PPV)—are used in dynamic assessments of fluid responsiveness [13].

Finally, per Guyton’s model, venous return is a key determinant of CO and depends on the pressure gradient between mean systemic filling pressure (Pmsf) and right atrial pressure [13]. Venous volume includes stressed volume (contributing to pressure and flow) and unstressed volume (i.e., the content of the splanchnic and large-caliber peripheral veins). Fluids administration increases stressed volume, thereby raising Pmsf and augmenting venous return [13].

## 3. Evaluating Preload Responsiveness

Historically, static indices such as central venous pressure (CVP) were used as surrogates for intravascular volume. However, CVP is influenced by various variables—such as intrathoracic pressure, venous tone, and ventricular compliance—and has been shown to correlate poorly with right ventricular end-diastolic volume or with a patient’s position on the Frank–Starling curve, particularly in critically ill populations [14,15].

Consequently, dynamic indices have gained prominence for guiding volume therapy. These indices, which either exploit heart–lung interactions or mimic transient fluid challenges, provide more reliable predictions of fluid responsiveness [16]. Several such tests are available [4]:

- **Fluid challenge** involves the rapid infusion of 500 mL of crystalloids over 10–15 min, to assess the resulting changes in CO. An increase in CO of ≥15% is generally considered indicative of fluid responsiveness. However, if the patient is not fluid-responsive, the administered volume cannot be withdrawn and may contribute to fluid overload [17].
- **Passive leg raising (PLR)** transiently transfers venous blood from the lower limbs to the central circulation, mimicking a reversible fluid challenge. A significant increase in SV and CO following PLR (≥10%) suggests fluid responsiveness [18].
- **Pulse pressure variation (PPV)** and stroke volume variation (SVV) rely on heart–lung interactions during positive pressure ventilation. They evaluate the cyclic changes in arterial pressure or stroke volume induced by mechanical breaths, serving as indicators of preload responsiveness. PPV and SVV values ≥ 12% indicate preload responsiveness [19].
- The **end-expiratory occlusion (EEXPO)** test requires briefly to interrupt mechanical ventilation at end expiration, thereby increasing venous return and preload. A subsequent rise in SV or CO ≥ 5% indicates preload responsiveness [20,21].
- The **PEEP test** consists in transiently decreasing the positive end-expiratory pressure (PEEP) to 5 cmH_2_O in mechanically ventilated patients with a PEEP ≥ 10 cmH_2_O; an increase in CO ≥ 8.6% reflects preload responsiveness [22].

All of these dynamic tests require real-time monitoring of cardiac output or stroke volume, which can be achieved using various techniques differing primarily in their level of invasiveness [4].

## 4. Monitoring Methods Without Specialized Equipment

Patient monitoring can be performed using simple, non-invasive techniques that do not require complex equipment, primarily relying on clinical observation and standardized assessment scales. These methods are particularly useful in emergency situations or when advanced devices are unavailable, leading to the creation of clinical scales that could help in stratifying patients according to severity of diseases [23]. Manual measurement of vital parameters such as heart rate, blood pressure, and respiratory rate remains of paramount importance, along with the observation of consciousness level, skin color, peripheral temperature, and urine output, which provide essential insights into hemodynamic status and tissue perfusion [24]. Assessment of heart rate is crucial for identifying compensatory mechanisms or underlying pathologies [24]. Additionally, clinical signs such as delayed capillary refill, cold sweating, and reduced urine output are important indicators of hypoperfusion and should guide therapeutic decisions [25]. Finally, monitoring respiratory rate and measuring oxygen saturation using basic pulse oximeters are vital parameters for evaluating both respiratory and cardiovascular status, particularly in patients with hemodynamic compromise [26]. Blood pressure, one of the key vital signs, results from the product of cardiac output and systemic vascular resistance. The latter plays a crucial role in characterizing the type of shock: reduced peripheral resistance typically indicates distributive shock—including anaphylactic, neurogenic, and septic shock—whereas increased resistance is commonly seen in cardiogenic shock. Cardiac output itself depends on stroke volume and heart rate; both parameters can be significantly affected during shock states. Notably, both extreme tachycardia and severe bradycardia can compromise cardiac output and contribute to hemodynamic instability [24].

Although knowledge of hemodynamic physiology and the use of clinical severity scales are valuable aids in clinical monitoring, they may still be subject to inaccuracies and inherent limitations. For this reason, wherever possible, it is always preferable to complement these observations with more advanced monitoring techniques that can provide more accurate and timely data for optimal patient management.

## 5. Invasive Monitoring Techniques

Invasive methods for CO measurement primarily include pulmonary artery thermodilution (PATD), transpulmonary thermodilution (TPTD), and lithium dilution techniques [27].

PATD involves the insertion of a pulmonary artery catheter (PAC) via central venous access. Cold fluid is injected into the right atrium, and the resulting temperature change is measured in the pulmonary artery to calculate right ventricular output. While accurate, PATD is invasive and carries risks such as thrombosis, hematoma, and infection. It is therefore not suitable for the Emergency Department setting [27].

TPTD uses a central venous catheter for cold saline injections and a femoral arterial catheter equipped with a thermistor to detect downstream temperature changes. The resulting thermodilution curve allows the estimation of CO as well as other advanced hemodynamic variables [28,29]. Although less invasive than PATD, its placement may be still impractical in the ED.

Lithium dilution requires the injection of lithium chloride through a central venous line. After traversing the pulmonary circulation, lithium concentration is measured in the arterial system using a lithium-sensitive electrode. Though generally well tolerated, repeated lithium measurements may pose a risk of accumulation, particularly in patients with impaired renal function [30].

Although these invasive techniques provide accurate hemodynamic data, they are associated with procedural risks and are typically reserved for critically ill patients requiring advanced monitoring in the Intensive Care Unit.

## 6. Non-Invasive Monitoring Technologies

In the ED, the need for rapid, accurate, and non-invasive hemodynamic assessment is essential for guiding fluid resuscitation and early management of acutely ill patients [27]. Several non-invasive monitoring techniques have emerged as valuable tools for assessing preload responsiveness. These methods are particularly well-suited for the ED environment, where time constraints, limited resources, and the need to avoid invasive procedures must be carefully balanced [27].

Among them, bioreactance-based CO monitoring, aortic velocity time integral (VTI) measurement via transthoracic echocardiography, non-invasive pulse contour analysis, and plethysmographic indices such as the perfusion index (PI) represent the most promising approaches [28,29,30]. Each modality has unique advantages and limitations, but shares the common benefit of being applicable at the point of care by emergency physicians, thereby enhancing early hemodynamic decision-making in unstable patients [27]. The characteristics of each modality are summarized in Table 1.

### 6.1. Bioreactance

Bioreactance (Starling, Baxter, Deerfield, IL, USA) is a non-invasive technology for continuous CO monitoring that detects phase shifts in an alternating current (AC) transmitted through the thorax [14]. These phase shifts are caused by pulsatile changes in thoracic blood volume during the cardiac cycle, which alter thoracic electrical conductivity [6]. As the AC signal traverses the chest, both voltage amplitude and a phase delay (the angular difference between voltage and current sine waves) are measured via four transmitting and four receiving electrodes placed on the chest wall.

The observed phase shifts are directly proportional to SV. The system calculates flow as the time derivative of the bioreactance signal (dBioreactance) and estimates SV using the formula:

SV = [dX/dt max] × VET

where dX/dt max represents the peak flow and VET (ventricular ejection time) is derived from the bioreactance waveform.

Cardiac output is then calculated as:

CO = SV × HR

with heart rate (HR) measured via chest sensors. The system also incorporates patient-specific parameters (e.g., age, weight, height, and body surface area) to refine its estimations [31].

Bioreactance offers continuous, operator-independent, and non-invasive CO and cardiac index monitoring, without the risks associated with invasive techniques [32,33]. Its portability and safety enable its use in a wide range of clinical settings, including spontaneously breathing patients, operating rooms, EDs, obstetric care, and dialysis units [34,35,36].

In particular, the reliability of this method in the emergency department context is supported by several studies. One example is a 2019 observational study that evaluated the use of bioreactance for monitoring cardiac output in non-ventilated patients in the emergency department who required volume resuscitation [27]. The study included 76 patients and compared bioreactance with other non-invasive monitoring techniques. The results highlighted that bioreactance was superior in terms of repeatability and operator independence, making it particularly useful in the emergency department setting, where patients are often in critical and confused states, due to its operator-independent nature. The technology demonstrated good reliability in the continuous monitoring of stroke volume (SV) and cardiac output (CO), with a strong correlation with other invasive techniques [27].

Additionally, another large study assessed the accuracy and precision of the NICOM (Non Invasive Cardiac Output Monitoring) bioreactance system in a cohort of 110 patients, with a total of 65,888 sample pairs. The results confirmed that the NICOM system demonstrated acceptable precision and high reactivity in detecting changes in CO across various clinical scenarios, including in emergency patients [37].

### 6.2. Point-of-Care Ultrasound (POCUS) for Hemodynamic Monitoring

Point-of-Care Ultrasound (POCUS) has gained increasing relevance as a rapid and versatile tool for hemodynamic assessment at the patient’s bedside, particularly in emergency and intensive care settings. It allows for real-time analysis of cardiac function, blood volume status, and fluid therapy response, without the need for advanced diagnostic facilities [38].

Through specific ultrasound windows, including parasternal, apical, and subcostal views in echocardiography, or by assessing the size and collapsibility of the inferior vena cava during respiration, POCUS can offer critical insights into preload status and the likelihood of response to fluid therapy. These parameters are essential for informing therapeutic decisions related to fluid resuscitation and the administration of vasoactive medications, particularly in hemodynamically unstable patients, where prompt intervention is paramount [39].

The ease of use and non-invasive nature of POCUS make it particularly well-suited for the emergency department setting, where timely diagnostic information can significantly impact clinical outcomes. Although image quality and interpretation depend on the operator’s experience, specific training programs and standardized protocols are improving the reliability and reproducibility of the technique [40].

The integration of POCUS with other non-invasive monitoring techniques contributes to a more accurate clinical assessment and supports a more personalized approach to hemodynamic management.

### 6.3. Transthoracic Echocardiography

**Figure 2:**
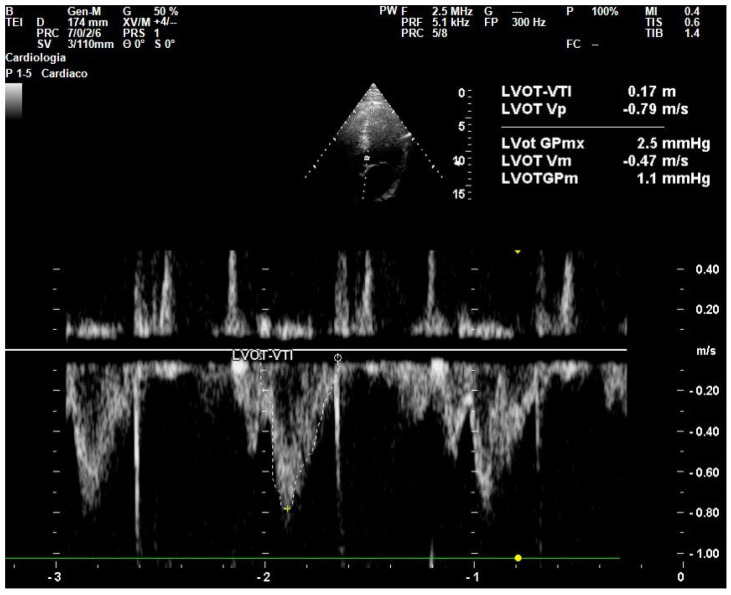
Example of Doppler with pulsed waves in the LVOT and measurement of the VTI tracing in apical 5-chamber echocardiographic view.

Transthoracic echocardiography (TTE) is a widely used indirect method for estimating CO using the VTI at the left ventricular outflow tract (LVOT) (Figure 2) [41]. Since CO originates at the LVOT, Doppler ultrasound can assess the velocity at which blood is ejected from the left ventricle. Stroke volume is estimated by using pulsed-wave Doppler echocardiography, based on the formula:

SV = LVOT Area ×LVOT VTI

where the LVOT area is calculated from the LVOT diameter (measured using the parasternal long-axis view, just below the aortic valve leaflets), and the VTI represents the area under the Doppler velocity curve obtained at the apical five-chamber view. VTI reflects the distance blood travels during systole and is averaged over three cardiac cycles for accuracy [42]. Normal LVOT diameters typically range between 1.8 and 2.3 cm and normal VTI values range from 18 to 25 cm.

These concepts can be summarized using the following formulas:

$$CI=\frac{CO}{BSA}=\frac{SV\times HR}{BSA}$$

$$CI=\frac{LVOT\;Area\times LVOT\;VTI\times HR}{BSA}$$

Studies have demonstrated that this technique is reliable in the emergency department setting, particularly in the assessment of patients with septic shock, where cardiac output (CO) monitoring is crucial to guide clinical management. Measurement aortic VTI has shown good concordance with invasive methods, with a high degree of repeatability even in non-ventilated patients and those in critical conditions [43]. Furthermore, the ability to monitor CO in real-time in a high-acuity environment has been considered particularly advantageous in the emergency department, to the point that it is regarded as a technique that every emergency physician should acquire [44].

### 6.4. Pulse Contour Analysis and Photoplethysmography

Pulse contour analysis and photoplethysmography are increasingly recognized as valuable non-invasive methods for hemodynamic monitoring in the ED [45]. These techniques provide rapid, bedside cardiovascular assessment without the need for invasive catheters, making them highly applicable in acute care setting [45].

Non-invasive pulse contour analysis systems (e.g., ClearSight—Edwards Lifesciences Corporation, Irvine, CA, USA; CNAP—CNSystems Medizintechnik GmbH, Graz, Austria) utilize finger cuff-based volume-clamp methods to continuously record arterial pressure waveforms. Proprietary algorithms are then used to estimate SV, CO and dynamic indices such as SVV [46]. Their ability to provide beat-to-beat hemodynamic data with minimal setup makes them suitable for assessing responses to preload-modifying maneuvers, even in spontaneously breathing patients [45].

Although slight differences have been observed compared to invasive methods, the bias between the two approaches has been considered acceptable, with the ClearSight system demonstrating rapid responsiveness in detecting hemodynamic fluctuations, making it useful for monitoring critically ill patients [47]. The reliability of these devices has also been confirmed in validation studies in the emergency department, where they have been used to risk-stratify patients with acute heart failure, thereby improving therapeutic management [48].

Photoplethysmography (PPG) commonly integrated into pulse oximeters, detects variations in light absorption determined by pulsatile arterial blood flow in peripheral tissues [49]. From the photoplethysmographic waveform, the perfusion index is calculated as the ratio between the pulsatile (arterial) and non-pulsatile (venous and tissue) components of the signal [49]. Although traditionally used as an indicator of peripheral perfusion, PI has gained attention as a surrogate for fluid responsiveness [18]. Dynamic changes in PI during preload-modifying maneuvers—such as PLR or EEXPO test—have shown to identify preload responsiveness [18,34,35]. Furthermore, a recent study has shown that PPG can be a useful tool not only for identifying the hemodynamic instability of patients in the emergency department [50], but also for identifying, in this context, those who may be affected by deep vein thrombosis (DVT) of the lower limbs, thereby improving the timeliness of diagnosis and treatment [51]. Whilst these technologies lack the precision of invasive gold standards, their ease of use, affordability, rapid deployment, and minimal operator dependence make them particularly advantageous in the ED [45]. When applied appropriately and with full awareness of their limitations, these non-invasive tools can enhance early individualized hemodynamic assessment in critically ill patients [45].

## 7. Emerging Technologies

Recent technological advances have enabled the development of wearable and portable monitoring systems capable of acquiring hemodynamic data in a non-invasive, continuous, and often wireless manner. These innovations enhance safety by eliminating catheter-related risks and allow the early detection of clinical deterioration, even outside the ICU settings [52].

One such device is the BioButton^®^ system (BioIntelliSense, Golden, CO, USA) which demonstrated promising results in a 2024 retrospective study involving 11,977 patients. The system continuously monitored over 20 vital parameters—including heart rate, respiratory rate, SpO_2_, and temperature—and significantly improved the early identification of clinical decline [53].

Wearable electrocardiographic devices and flexible photoplethysmographic sensors are also gaining traction. PPG sensors detect blood volume changes in microvascular tissues using light-emitting diodes (LEDs) and photodiodes, enabling non-invasive estimation of blood pressure and volume status. The flexibility of these sensors allows placement on various body sites and supports continuous, motion-tolerant monitoring [54]. Notably, a study published by Lee et al. demonstrated that a polarized light-based flexible PPG sensor maintained signal fidelity even during patient movement, underscoring its potential for use in dynamic clinical settings [55]. In this regard, VitalStream^®^ (Caretaker Medical, Charlottesville, VA, USA) is currently the only FDA-approved wearable and wireless hemodynamic monitoring device utilizing PPG. It provides continuous measurement of parameters such as heart rate and blood pressure and incorporates artificial intelligence (AI) for real-time data interpretation. VitalStream^®^ demonstrated clinical accuracy comparable to pulmonary artery catheter measurements in cardiac surgery patients and its use has been associated with enhanced patient mobilization and reduced ICU stay and complications [56,57]. A 2024 study examined the use of VitalStream^®^ in the emergency department setting, demonstrating that the device is capable of rapidly detecting changes in patients’ clinical conditions, such as variations in blood pressure and heart rate. Continuous, non-invasive monitoring allows for timely management of patients, reducing the risk of complications in critically ill patients and improving early mobilization during ICU admission, as highlighted by previous studies [58].

This technology has the potential to transform patient management in the emergency department, enabling more precise and personalized monitoring of hemodynamic conditions, with particular benefit for patients in unstable or rapidly changing states. While these technologies offer advantages—including real-time surveillance, early warning capability, and potential reduction in morbidity and mortality—their widespread adoption faces keys challenges. These include ensuring sensor accuracy, optimizing power efficiency, managing data security and interoperability, and integrating seamlessly with existing hospital information systems. The incorporation of AI represents a significant step forward, enabling predictive analytics and proactive clinical decision-making rather than passive data collection. Nonetheless, large-scale validation studies are needed to confirm their safety, accuracy, and cost-effectiveness across diverse patient populations and care settings [59].

## 8. Advantages and Limits in the ED Setting

The increasing adoption of non-invasive hemodynamic monitoring systems in the ED reflects their growing role in the early management of critically ill patients. These technologies offer significant advantages, including rapid deployment, operational simplicity, and a substantially lower risk of procedure-related complications compared to invasive ones [60,61]. In high-acuity environments, the ability to obtain real-time hemodynamic data quickly is essential. Non-invasive systems enable early assessment of circulatory status, facilitating prompt diagnosis and timely intervention in time-sensitive conditions such as major trauma, septic shock, or acute myocardial infarction [62,63]. Their ease of use and minimal training requirements make them particularly well-suited for integration into emergency protocols, even by non-specialist personnel.

A further strength of these systems lies in their capacity for continuous, real-time monitoring of key physiological parameters—including heart rate, arterial pressure, and oxygen saturation—without the need for vascular access. Technologies such as light reflectance plethysmography or bioreactance provide dynamic tracking of clinical trends, which is particularly beneficial in patients with early hemodynamic instability or evolving pathology [62,63]. Most importantly, the non-invasive nature of these tools significantly reduces the risk of complications such as infection, bleeding, and thrombosis—not an irrelevant consideration for unstable or immunocompromised patients [46]. The ability to collect clinically useful data quickly and safely supports more responsive decision-making and may contribute to improved clinical outcomes.

Despite these numerous advantages, non-invasive systems have limitations that must be acknowledged. Their accuracy remains inferior to that of invasive techniques, particularly in cases of marked hemodynamic instability—such as hypovolemic or cardiogenic shock—where altered blood flow and peripheral vasoconstriction can compromise signal integrity [64,65,66]. These systems rely on algorithmic interpretation of physiological signals, making them vulnerable to interference from patient-related factors such as obesity, generalized edema, reduced peripheral perfusion, and arrhythmias (e.g., atrial fibrillation), which disrupt the underlying assumptions of regular cardiac rhythm [67,68,69]. Additional challenges include susceptibility to motion artifacts, environmental interference, suboptimal skin contact, and dermal conditions which can affect data accuracy and reliability [70,71,72,73,74]. Furthermore, the lack of real-time calibration in many devices can limit measurement precision during resuscitation or transport scenarios [70]. Most non-invasive devices also lack the capacity to measure advanced hemodynamic indices such as pulmonary capillary wedge pressure, central venous oxygen saturation, or precise continuous cardiac output, which remain critical in the management of complex critical pathologies [48,75].

## 9. Future Perspectives and Technological Innovation in Non-Invasive Monitoring

Technological innovation and research are transforming non-invasive monitoring systems in emergency and intensive care medicine. A significant advancement is the development of advanced sensors that measure complex hemodynamic parameters without the need for invasive catheterization. These technologies allow for rapid and accurate assessments, improving the decision-making process, especially in critical situations such as septic or cardiogenic shock [76].

Furthermore, the use of artificial intelligence (AI) and machine learning represents another step forward in non-invasive monitoring. These technologies process large volumes of patient data to detect early warning signs and generate real-time alerts. In the emergency department, for example, AI can simultaneously monitor multiple patients and identify deviations from normal parameters, assisting clinical staff in making timely decisions [77].

Despite these advancements, challenges remain concerning the reliability of systems, particularly during acute hemodynamic instability. The accuracy of some techniques may be compromised by external factors in acute shock situations [67].

In summary, non-invasive monitoring systems offer a promising future for emergency and intensive care medicine. Technological advancements will enhance the speed and accuracy of assessments, reducing the risks associated with invasive procedures. Future research, combined with the integration of advanced technologies, is likely to lead to more personalized monitoring and targeted therapeutic approaches. As these systems evolve, non-invasive monitoring will become a fundamental element in emergency care, improving treatment effectiveness and overall care quality.

## 10. Conclusions

Non-invasive monitoring technologies represent a promising frontier in emergency medicine. With ongoing innovations, they are expected to enhance the speed, accuracy and safety of hemodynamic assessment, while supporting more personalized, physiologically-guided therapeutic strategies. As these tools continue to evolve, they are likely to become integral components of emergency care, contributing to improved patient outcomes and more efficient resource utilization. However, their effective application requires clinicians to interpret each parameter within its pathophysiological context, ensuring that technological advances are matched by clinical judgment.

Among the techniques discussed, Point-of-Care Ultrasound (POCUS) stands out for its ability to provide a rapid, bedside, and multimodal assessment of cardiac function and volume status, particularly useful in hemodynamically unstable patients. Complementary to POCUS, bioreactance and pulse contour analysis offer continuous and operator-independent monitoring, facilitating dynamic assessment of fluid therapy response in the emergency department setting, although with limitations in cases of severe hypoperfusion or arrhythmias.

Emerging wearable technologies, leveraging flexible photoplethysmography and artificial intelligence, show potential for continuous, non-invasive, and predictive hemodynamic monitoring. However, large-scale clinical validation is required before widespread adoption.

In conclusion, no single technique can be considered a gold standard in isolation. A comprehensive approach that integrates POCUS with other continuous non-invasive methods is currently the most effective strategy for personalized and timely hemodynamic monitoring in emergency situations. The selection of monitoring tools should be adapted to the patient’s specific needs, clinical urgency, and the experience of the healthcare provider, with the goal of optimizing fluid management and enhancing clinical outcomes.

## Figures and Tables

**Table 1:** Summary of the characteristics of non-invasive monitoring technologies.

Technique	Advantages	Disadvantages	Available Parameters	Procedural Requirements	Cost
**Bioreactance**	- Continuous, non-invasive CO and cardiac index monitoring - Operator-independent - Portable and safe - Applicable in various clinical settings	- Limited accuracy in patients’ movement, electrode poor skin contact - Less precise than invasive methods	CO, cardiac index, stroke volume (SV)	Application of thoracic electrodes	Moderate to high
**Transthoracic Echocardiography (TTE)**	- Real-time, multimodal assessment of cardiac function and volume status - Guides immediate therapeutic decisions - Detailed SV and VTI measurement	- Operator-dependent - Requires specific training - Intermittent rather than continuous monitoring	VTI, SV, CO, ventricular function	Portable ultrasound device, trained operator	Moderate to high
**Pulse Contour Analysis & Photoplethysmography (PPG)**	- Continuous, rapid, non-invasive monitoring - Low operator dependency - Often integrated into standard devices (e.g., pulse oximeters)	- Limited accuracy in severe hypoperfusion, arrhythmias or vasoconstriction	SV, CO, dynamic indices (e.g., SVV), perfusion index (PI), dynamic changes in PI	Finger cuff device or pulse oximeter with integrated sensor	Low to moderate

## Data Availability

Not applicable.

## Abbreviations

The following abbreviations are used in this manuscript:

AC	Alternating Current
AI	Artificial Intelligence
CI	Cardiac Index
CO	Cardiac Output
CVP	Central Venous Pressure
DBP	Diastolic Blood Pressure
DVT	Deep Vein Thrombosis
EEXPO	End-Expiratory Occlusion
ED	Emergency Department
EDV	End-Diastolic Volume
ESV	End-Systolic Volume
HR	Heart Rate
ICU	Intensive Care Unit
LVOT	Left Ventricular Outflow Tract
MAP	Mean Arterial Pressure
NACA	National Advisory Committee for Aeronautics
NICOM	Non-Invasive Cardiac Output Monitoring
PAC	Pulmonary Artery Catheter
PATD	Pulmonary Artery Thermodilution
PCWP	Pulmonary Capillary Wedge Pressure
PEEP	Positive End-Expiratory Pressure
PI	Perfusion Index
PLR	Passive Leg Raising
POCUS	Point-of-Care Ultrasound
PP	Pulse Pressure
PPG	Photoplethysmography
PPV	Pulse Pressure Variation
Pmsf	Mean Systemic Filling Pressure
SBP	Systolic Blood Pressure
SV	Stroke Volume
SVV	Stroke Volume Variation
TPTD	Transpulmonary Thermodilution
TTE	Transthoracic Echocardiography
VET	Ventricular Ejection Time
VTI	Velocity Time Integral
